# Genome Sequence of Pantoea ananatis SGAir0210, Isolated from Outdoor Air in Singapore

**DOI:** 10.1128/genomeA.00643-18

**Published:** 2018-07-05

**Authors:** Irvan Luhung, Ana Carolina M. Junqueira, Akira Uchida, Rikky W. Purbojati, James N. I. Houghton, Caroline Chénard, Anthony Wong, Megan E. Clare, Kavita K. Kushwaha, Deepa Panicker, Alexander Putra, Nicolas E. Gaultier, Balakrishnan N. V. Premkrishnan, Cassie E. Heinle, Vineeth Kodengil Vettath, Daniela I. Drautz-Moses, Stephan C. Schuster

**Affiliations:** a Singapore Centre for Environmental Life Sciences Engineering, Nanyang Technological University, Singapore; b Departamento de Genetica, Instituto de Biologia, Universidade Federal do Rio de Janeiro, Rio de Janeiro, Brazil; c Asian School of Environment, Nanyang Technological University, Singapore

## Abstract

Pantoea ananatis SGAir0210 was isolated from outdoor air collected in Singapore. The genome was assembled from long reads generated by single-molecule real-time sequencing complemented with short reads. The genome size was approximately 4.81 Mb, with 4,303 protein-coding genes, 80 tRNAs, and 22 rRNAs identified.

## Abstract (precis)

Pantoea ananatis SGAir0210 was isolated from outdoor air collected in Singapore. The genome was assembled from long reads generated by single-molecule real-time sequencing complemented with short reads.

## GENOME ANNOUNCEMENT

The Gram-negative bacterium Pantoea ananatis belongs to the family Enterobacteriaceae within the phylum Proteobacteria. This bacterium has been isolated from various habitats, such as plants (1), soil (2), water (3), and aviation fuel tanks (4). While there have been reports of human infection (5, 6), P. ananatis is known primarily as a pathogen in plant hosts, such as rice (7), maize (8), and onion (9). Recent findings, however, have also highlighted the existence of beneficial strains that live on the hosts as commensals or plant growth promoters (10–12).

Here, we present the genome of P. ananatis SGAir0210, isolated from tropical air by means of air sampling at an outdoor location in Singapore (1.350°N, 103.689°E) using an Andersen single-stage impactor (SKC BioStage). Air was drawn at a 28.3 liter/min flow rate and directly impacted onto marine agar (Becton, Dickinson) that was mounted on the sampler for 4 min. After incubation at 30°C, a colony was replicated in Trypticase soy agar to isolate a single organism. The pure culture was finally grown in Luria-Bertani broth (30°C) overnight before DNA extraction.

DNA was extracted using a Wizard genomic DNA purification kit (Promega) following the standard protocol. After extraction, sequencing was conducted on a Pacific Biosciences RS II platform utilizing three single-molecule real-time (SMRT) cells and a SMRTbell version 1.0 template prep kit for library preparation. In addition, 300-bp paired-end sequencing was carried out on the Illumina MiSeq platform after library preparation using a TruSeq Nano DNA kit. The SMRT sequencing yielded 35,581 subreads, whereas the MiSeq run yielded 874,001 reads.

The genome was *de novo* assembled using the Hierarchical Genome Assembly Process (HGAP) version 3 (13) in the PacBio SMRT Analysis version 2.3.0 package. Final polishing and error correction were performed using the MiSeq paired-end reads with Quiver and Pilon version 1.16 (14), respectively. The assembly produced two contigs with a total size of 4,808,586 bp. The chromosomal contig had a size of 4,504,557 bp (57.2-fold coverage, 53.5% G+C content), while the plasmid contig was 304,029 bp long (44.6-fold coverage, 52.0% G+C content). The chromosomal contig was unable to be circularized via Circlator (15). Species identification using average nucleotide identity analysis (ANI) performed with MiSI (Microbial Species Identifier) (16) showed a 97.2% match to P. ananatis strain LMG 2665. Additional analysis with Phyla-AMPHORA identified 98.4% marker similarity to the genus Pantoea with minimum confidence of 1.0 (17).

Genome annotation was completed with the NCBI Prokaryotic Genome Annotation Pipeline (PGAP) version 4.2 (18). The annotation identified 4,589 genes, which consisted of 4,303 protein-coding genes, 121 RNAs (80 tRNAs, 19 noncoding RNAs, 8 subunits of the 5S rRNA, and 7 copies each of the 16S and 23S rRNAs), and 165 pseudogenes. Functional annotation with the Rapid Annotations using Subsystems Technology (RAST) server (19) identified 4,626 DNA coding sequences within 527 subsystems. Of these, 164 genes were annotated as genes related to stress response, such as heat and cold shock, which could be useful for airborne survival. Moreover, 40 genes were found to be related to phages and prophages, including proteins that are part of the phage tail and replication.

### Accession number(s).

The genome sequence of P. ananatis SGAir0210 has been deposited in DDBJ/EMBL/GenBank under the accession numbers CP028033 to CP028034.
